# Are We Facing a New Colposcopic Practice in the HPV Vaccination Era? Opportunities, Challenges, and New Perspectives

**DOI:** 10.3390/vaccines9101081

**Published:** 2021-09-26

**Authors:** Ankica Lukic, Rosa De Vincenzo, Andrea Ciavattini, Caterina Ricci, Roberto Senatori, Ilary Ruscito, Antonio Frega

**Affiliations:** 1 Department of Surgical and Medical Sciences and Translational Medicine, Sapienza University of Rome, Sant’Andrea Hospital, Via di Grottarossa 1035-1039, 00189 Rome, Italy; ankica.lukic@uniroma1.it (A.L.); ilary.ruscito@uniroma1.it (I.R.); antonio.frega@uniroma1.it (A.F.); 2 Italian Society of Colposcopy and Cervicovaginal Pathology (SICPCV), 00186 Rome, Italy; rosa.devincenzo@unicatt.it (R.D.V.); andrea.ciavattini@ospedaliriuniti.marche.it (A.C.); robertosenatori@gmail.com (R.S.); 3 Gynecologic Oncology Unit, Fondazione Policlinico Universitario A. Gemelli, IRCCS, Dipartimento Scienze della Salute della Donna, del Bambino e di Sanità Pubblica, 00168 Rome, Italy; 4 Dipartimento di Scienze della Vita e Sanità Pubblica, Università Cattolica del Sacro Cuore, 00168 Rome, Italy; 5 Gynecologic Section, Department of Odontostomatologic and Specialized Clinical Sciencies, Università Politecnica delle Marche, 60121 Ancona, Italy

**Keywords:** colposcopy, HPV vaccines, high grade intraepithelial lesions, cervical cancer, cervical adenocarcinoma, risk stratification, HPV genotyping, colposcopic referral criteria

## Abstract

The combination of primary and secondary prevention has already influenced the colposcopic practice by reduction in HPV (human papillomavirus) vaccine-type HSIL (HIGH-GRADE SIL), colposcopy referral numbers, colposcopic positive predictive value (PPV) for CIN2+, and by modification of referral pattern, colposcopic performance, and procedures. Different strategies, both isolated and combined, have been proposed in order to maintain the diagnostic accuracy of colposcopy: patient risk stratification based on immediate or future risk of CIN3+ or on HPV genotyping after a positive screening test. Data are needed to support alternative colposcopic strategies based on vaccination status and on the application of artificial intelligence where the patient’s risk stratification is implicit in precision medicine which involves the transition from an operator-dependent morphology-based to a less-operator dependent, more biomolecular management. The patient’s risk stratification based on any combination of “history” and “test results” to decrease colposcopy workload further reduce colposcopic and histologic morphological approaches, while adding genotyping to the risk stratification paradigm means less cytologic morphologic diagnosis. In Italy, there is a strong colposcopic tradition and there is currently no immediate need to reduce the number of colposcopies. Instead, there is a need for more accredited colposcopists to maintain the diagnostic accuracy of colposcopy in the vaccination era.

## 1. Introduction

The World Health Organization (WHO) has called for global action towards the elimination of cervical cancer by 2030 until it becomes a rare tumor [1,2]. This project will probably be achieved through the impact of primary and secondary prevention and will take several decades to reach the predetermined goal, i.e., less than four new cases of cervical cancer per 100,000 women [2]. We are experiencing a real-life revolution in the prevention of cervicocarcinoma and the changes in colposcopic practice are essentially secondary to primary and secondary strategies. Primary prevention using vaccination against high oncogenic-risk human papillomavirus (HPV) certainly represents one of the most important achievements in the field of medicine in the last 20 years, that brings advantages in terms of global health and quality of life of vaccinated subjects. The prophylactic efficacy of the vaccines has been assessed on the basis of data from randomized controlled trials. To date, we have long-term immunogenicity, effectiveness, and safety data in young women that arrive at 12–14 years of follow-up for quadrivalent [3] and 6–8 years following vaccination of girls and boys aged 9–15 years with nonvalent vaccine [4]. Obviously, the duration of protection against vaccine genotypes has not been established, despite this, it was assumed for a lifetime. In the systematic review of 10 years of impact and effectiveness of the quadrivalent vaccine in the real-life world experience, a reduction of 90% of HPV 6/11/16/18 infection and 90% of genital warts, of 45% of low-grade SIL, and 85% of histologically proven high-grade SIL (HSIL CIN2, CIN3) has been reported [5]. The nine-valent HPV vaccine, which was approved in Europe in June 2015, is expected to prevent up to 90% of cervical cancer (compared to 70% of the other two vaccines), and up to 96% of anal cancers [6]. The recommended dosing schedule is two doses for the younger population (9–15 years) and three doses for older subjects. In this scenario, the HPV vaccines have essentially four characteristic aspects to consider (Box 1).

Box 1 **HPV vaccines.**

- HPV vaccines are prophylactic;
- population coverage is not yet as expected (95%);
- vaccines do not protect against all HPV types that cause precancer and cancer, although the nine genotypes covered account for 90% of cancers;
- duration of vaccine protection and long-term efficacy is not known, but at least 12 years has already been demonstrated and lifetime protection assumed;
- vaccination effectiveness seems to be related to the interval of time between the first vaccination and the screening program. Younger age at immunization is associated with increasing vaccine effectiveness.

The interval of time between vaccination and screening should be considered in future screening and follow-up algorithms, as it is notable that the HPV vaccine effectiveness increases with increasing intervals between first vaccination and earliest detection test [7]. The effect of HPV vaccination strongly depends on both the target population and the rate of vaccination coverage. The vaccine coverage among female teenagers aged 10–20 years varies enormously from 1.2% to 53% in different populations (53% in North America, 41% in Oceania, 36% in Europe, 22% in Latin America and the Caribbean, and 1.2% in Africa and Asia) [8]. In Europe, it accounts for around 36%, in Italy about 40%, where the vaccine coverage of the 2006 cohort ranges from 17 to 71% in different regions [8]. It is clearly established that only a high vaccination coverage will allow the screening algorithm modification with screening interval elongation. We are all convinced that, even in the most optimistic real-life settings, it is unlikely that optimal vaccination coverage will be achieved soon in different regions and different countries. On the other hand, even the year of initiation of the national HPV vaccination program, just as of the starting year, the screening age, the interval, and the primary test used in the national screening programs really differ in the different geographical areas [9]. As we know, for all these reasons, cervical cancer screening in the vaccination era will have to continue for decades to account for non-vaccinated/partially vaccinated cohorts. There will inevitably be a differentiated screening strategy between vaccinated and unvaccinated cohorts. However, the ethical relevance of primary prevention is not limited only to the efficacy, safety, immunogenicity, and tolerability profile of the vaccines, but also includes social and economic costs [10]. In Italy, the total cost of the annual incident cases of the nine-valent HPV vaccine was estimated to be EUR 528.6 million [9]. Therefore, vaccination with nine-valent vaccines will lead to a significant reduction in healthcare costs in terms of diagnosis, treatment, and follow-up. Most of the HPV vaccination impact and effect is essentially measured as post-vaccination changes of viral genotypes prevalence, and abnormal cytology, genital warts, intraepithelial lesions, and recently, invasive cervical neoplasms incidence [11,12,13]. Now, we indeed need more numerous vaccine impact studies focused on colposcopy practice. Colposcopy with targeted biopsy will remain for decades the primary method of detecting precancers requiring treatment. 

## 2. Referral Criteria for Colposcopy in the Vaccination Era: Changes and Challenges

The demand for colposcopy services is influenced by a number of factors, first of all, by the referral criteria as a result of the target screening population and the primary screening test used.

### 2.1. Target Screening Populations and Colposcopy in the Vaccination Era 

Different target screening populations characterized by a different vaccination status are commonly referred to as colposcopy in the present era [14,15,16] (Box 2):

- cohorts not offered vaccination;
- vaccinated girls and women with different vaccine and at different age;
- unvaccinated girls and women within vaccinated cohorts (at different ages and with different vaccines);
- vaccinated cohorts in whom it is not known who has and has not been vaccinated and with which vaccine.
- women vaccinated across or after treatment for HSIL with an adjuvant intent.

Box 2 **Issues in target screening populations, primary screening tests, and colposcopy in the vaccination era.**

**Screening population:**

- Different vaccination status of the population;
- different age cohorts in vaccinated and screened population;
- different timing between vaccination and screening test;
- different vaccine effectiveness;

**Primary screening test:**

- Different primary screening tests according to target population age and vaccination status;
- HR HPV partial genotyping as the primary screening test is used to refer only the highest risk group HPV 16/18 positive to direct colposcopy;
- HR HPV extended genotyping as an additional or primary screening test repre-sents; an essential step of HPV-risk estimation even if the additional risk strati-fication needs further evidence.

Obviously, such a diversity of the screening populations does not facilitate a univocal approach to all the stages of the prevention process, included colposcopy. Indeed, only a universal vaccination strategy will, over time, lead to greater homogeneity of the populations to be screened. In the female population, considering the age of vaccine administration, we can identify at least three vaccinated cohorts: young girls vaccinated at 12 years or earlier, catch-up vaccination within 25 years, and vaccination after 25 years. Before several cohorts are intensively vaccinated, discrimination of screening protocols as a function of the vaccination status of the individuals may appear to be the possible strategy in most settings. In fact, the “HPV vaccination status”, which has not yet been included in the recent clinical guidelines, will undoubtedly affect recommendations and guidelines about the management of positive screening tests [17]. At the moment, there is no general agreement that tailored screening protocols based on the vaccination status of the target population should replace the actual “one size fits all” protocol before high vaccination coverage and herd immunity effects in real life have been reached [15]. Only continuous monitoring of early on vaccinated cohorts will allow us to adapt the screening programs. 

### 2.2. Primary Screening Test and Colposcopy in the Vaccination Era 

When we consider the primary screening test, we are aware that we will deal, for some time, with women screened in different ways: only by primary cytology, by HR HPV test and triage cytology, by co-testing, and, in some settings, even by partial or extended genotyping.

Based on scientific evidence as well as national and international guidelines, organized screening programs in Italy have gradually introduced the HPV test as the primary screening test, replacing cytology in older women [18,19]. As in other countries, the current strategy is leading to a reduction in the incidence and mortality of cervical cancer in the Italian population (Box 2).

In settings where co-testing is used as a primary screening tool, the modifications of recommendations have recently been proposed: the ASCCP published new management guidelines for cervical cancer screening abnormalities, where paradigm shifts were proposed from result-based to risk-based guidelines [17]. Clearly defined risk thresholds to guide management are designed when the population-level prevalence of CIN3+ decreases after HPV vaccination. The clinical value and role of partial, extended, or full HR HPV genotyping as the triage following positive primary HR HPV screening testing in the vaccination era is still debated. Increasing evidence suggests that extended HPV genotyping (beyond 16/18) is effective for risk stratification even in women with normal cytology. Thus, the partial genotyping that distinguishes the positivity for HPV 16/18 from other HR HPV could also be used to refer only the highest risk group of patients to direct colposcopy. The addition of HPV 31 to HPV 16/18, as part of a primary HPV screening algorithm recommendation for colposcopy, offers potential benefits for the detection of high-grade cervical disease in the NILM cytology population [20]. Human papillomavirus 16 consistently carried the highest risk for CIN3+ (approximately 15–35% for any cytology and approximately 8–25% for normal cytology), HPV 31, 18, and 33 carried intermediate-high risk for CIN3+ (ranging from approximately 8% to 20% in any cytology and approximately 5% to 10% in normal cytology), HPV 52, 58, and 45 carried moderate risks, with HPV 35, 39, 51, 56, 59, 66, and 68 consistently having the lowest CIN3+ rates, regardless of cytology [20,21,22,23]. While recent studies highlight the possible role of HPV genotyping for clinical management of abnormal screening results, further evidence is still needed to assess both, the value of genotyping in different clinical settings (screening, diagnosis, treatment, and follow-up) and the additional risk stratification of more detailed genotyping. Optimal stratification of genotypes into risk levels is a promising advance but is feasible only using clinically validated extended genotyping HPV assays that mesh with clinical action thresholds [20,21,22,23,24]. The search for a new test or a combination of tests stands as a priority for the future. It has recently been emphasized that “it’s not the test you take but the decision you make” being pivotal [25]. This novel concept, that we all share, indicates the futuristic vision of the screening that is: “not only the tests one takes but the individual risk it makes”!

## 3. Colposcopic Practice in the Post-Vaccination Era 

Before the vaccination era, for around one century, colposcopy was performed right away in patients with abnormal cytology. The transition from Pap smear to HPV primary screening test already led to a decreasing trend in the number of referrals to colposcopy. New screening guidelines recommend colposcopy only when lesions are more likely to be detected (Box 3). Though colposcopy has made an undeniable contribution to the prevention and treatment of cervical precancer and cancer, its diagnostic accuracy in the vaccination era is more than ever unpredictable. We know that colposcopic accuracy is associated with variable sensitivity for HSIL CIN2+ that is reported to be 30–70% [26,27,28,29,30,31]. Since both bivalent and quadrivalent HPV vaccines target genotypes 16 and 18, the earliest changes in the screened vaccinated population involve lesions correlated to these two viral types. In fact, in the last 15 years, the reduction in vaccine type, HPV prevalence, and disease has been reported in many countries (Australia, USA, Scotland, France, New Zealand, Israel, Italy, etc.)—the impacts of which are already reducing the number of second-level colposcopic examinations.

Box 3 **Colposcopic practice in the vaccination era.**

HPV vaccination:

- Reduced the prevalence of HPV 16 (and other vaccine HPV types) infection and HPV 16 CIN2+;
- reduced the PPV of colposcopy for the detection of CIN2+;
- does not have a significant effect on commonly recognized colposcopic features; multiple, as opposed to single HR HPV infection, is associated with larger colposcopic lesions;
- modified pattern of referral to colposcopy: the proportion of LSIL increase, the proportion of HSIL decrease and therefore the number of referrals to colposcopy for HSIL decrease;
- modified colposcopic performance (increase in cases with absence of abnormal colposcopic features or those having no clinical interventions; decrease in the proportion of women with a colposcopic impression of high-grade SIL and those having diagnostic punch biopsy/biopsies or treatment) thus diagnostic and therapeutic procedures require major knowledge and skills;
- new screening guidelines recommend colposcopy only when lesions will likely be detected;
- modifications of colposcopic standards are required (more or less than 50 colposcopies/year for HSIL);
- higher-quality colposcopy service represents an essential component of future cervical cancer screening programs;
- new guidelines are moving from more operator-dependent morphology-based screening and diagnosis to less operator-dependent biomolecular management.

### 3.1. Reduction of HPV Vaccine-Types Prevalence and Disease in Italy

A randomized trial run in Italy suggests that HPV vaccination at the age of 25 years is beneficial if offered to HR HPV-negative women [32]. The real-life population effect of the quadrivalent vaccination programs on an 18–50 years old Italian cohort showed high effectiveness on vaccine-type HPV prevalence (standardized four-valent HPV vaccine type prevalence was 0.6% in vaccinated versus 5.5% in unvaccinated women, *p* < 0.001), and found no evidence of type-replacement [33]. In a retrospective cohort study carried out on women vaccinated by bivalent or quadrivalent vaccine diagnosed with HSIL (CIN2+) in four Italian centers between 2015 and 2017, the nine-valent HPV vaccine type was diagnosed in 81.8% of cases. Therefore nonvalent vaccination should theoretically improve protection against more than 80% of HPV-related lesions compared to other vaccines [34]. In the organized cervical screening program in Italy, catch-up HPV vaccination almost halved the risk of cytological abnormalities. Women receiving at least one bivalent or quadrivalent vaccine dose were significantly less likely to have abnormal cytology (adjusted odds ratio 0.52; 95% confidence interval 0.34–0.79) [35].

### 3.2. Reduction of HPV Vaccine-Types Squamous Disease (CIN2+) in Different Countries

In this scenario, the sentinel surveillance system in the USA reported a 26% reduction in HPV 16/18 associated CIN2+ following HPV vaccination and increasing HPV vaccine effectiveness with increasing interval between first vaccination and earliest detection of cervical disease [7]. From 2008 to 2012, the prevalence of HPV 16/18 CIN2+ lesions significantly decreased from 53.6% to 28.4% among women who received at least one vaccine dose but not among unvaccinated women or women with unknown vaccination status. Estimated vaccine effectiveness for prevention of HPV 16/18-attributable CIN2+ was 21% (95% CI: 1–37), 49% (95% CI: 28–64), and 72% (95% CI: 45–86) in women who initiated vaccination 25–36 months, 37–48 months, and >48 months prior to the screening test that led to CIN2+ diagnosis [7]. Since the introduction of HPV vaccination in Australia, women aged 12–26 years have shown a decrease of 34% in low-grade lesions and 47% in high-grade lesions at 5 years post-vaccination [36]. Smith et al. reported a similar reduction in the incidence of cervical dysplasia (44%) among girls aged 14 to 17 years in Canada [37]. Furthermore, routine vaccination of girls aged 12–13 years with the bivalent HPV vaccine in Scotland led to a dramatic reduction in preinvasive cervical disease at age 20 years. Younger age at immunization was associated with increasing vaccine effectiveness for CIN3+: 86% (75% to 92%) for women vaccinated at age 12–13 compared with 51% (28% to 66%) for women vaccinated at age 17. Evidence of herd protection against high-grade cervical disease was found in unvaccinated girls [38]. A significant reduction in cervical intraepithelial neoplasia CIN1, CIN2, and CIN3 (of 29%, 50%, and 55%, respectively) in Scottish women aged 20–21 was associated with three doses of bivalent HPV vaccine administered during a catch-up campaign for those under the age of 18 compared with unvaccinated women [39]. Further, in the group of young women with abnormal cytology referred to colposcopy, HPV vaccination via a catch-up program reduced the prevalence of CIN2+ and HPV 16 infection in Scotland. Though the HPV vaccine did not have a statistically significant effect on commonly recognized colposcopic features, there was a slight non-significant reduction in the positive predictive value (PPV) of colposcopy for CIN2+, from 74% (unvaccinated) to 66.7% (vaccinated). The presence or absence of HPV 16 had a significant impact on the specificity and NPV of colposcopy for detecting CIN2+ [40]. The correlation between vaccine efficacy, the time elapsed from vaccine administration, and the target population is further confirmed in the updated systematic review and meta-analysis by Dorlet et al. who reported that after 5–9 years of vaccination, CIN2+ decreased significantly by 51% (RR 0.49, 95% CI 0.42–0.58) among screened girls aged 15–19 years and decreased significantly by 31% (RR 0.69, 95% CI 0.57–0.84) among women aged 20–24 years [40]. Regarding preliminary data on invasive cervical cancer, interesting data from the U.S. reported that the incidence of cervical cancer was markedly decreased in the vaccine era compared with the pre-vaccine era only among young females aged 15–24 years where the 4-year average annual incidence rates for cervical cancer in 2011–2014 were 29% lower than that in 2003–2006 (6.0 vs. 8.4 per 1,000,000 people, rate ratio = 0.71, 95% CI = 0.64, 0.80) [11]. Moreover, among Swedish girls and women 10 to 30 years old, quadrivalent HPV vaccination was associated with a substantially reduced risk of invasive cervical cancer at the population level. After adjustment for age at follow-up, the incidence rate ratio for the comparison of the vaccinated population with the unvaccinated population was 0.51 (95% confidence interval [CI], 0.32 to 0.82). The importance of the age of vaccine administration was further underlined: the incidence rate ratio was 0.12 (95% CI, 0.00 to 0.34) among women who had been vaccinated before the age of 17 years and 0.47 (95% CI, 0.27 to 0.75) among women who had been vaccinated at the age of 17 to 30 years [12]. Since HPV vaccine introduction in U.S., cervical squamous cell carcinoma decreased by 22.5% per year and adenocarcinoma incidence rates declined by 9.4% per year among women aged 15–20 years according to population-based cancer registry data, possibly because of HPV vaccine impact [13].

### 3.3. Adenocarcinoma of the Cervix: Post Vaccination Incidence and Colposcopic Practice

In the era of increasing coverage of cervical cancer screening and HPV vaccines, cervical cancer incidence reduction primarily occurred in squamous cell cancers, with limited reductions in adenocarcinomas [12,41]. In the USA, the overall incidence of squamous cell cancer decreased annually between 1973 and 2007 by 8%, while the incidence of adenocarcinoma increased by an average of 2.9% per year over the same period [42]. The natural history of adenocarcinoma of the cervix differs from that of its squamous counterpart. Glandular precancerous lesions typically develop within the endocervical canal: therefore, they usually have a lower detection rate during routine cervical cytologic examination [43].

Moreover, colposcopic suspicion of glandular lesions is more difficult to reach, since the absence of characteristic colposcopic patterns. Endocervical adenocarcinomas are a heterogeneous group of tumors with varying etiologies, morphologies, biological and molecular signatures, and prognoses, accounting for approximately 25% of all cervical cancers [44]. Unlike squamous cell carcinomas, it is now evident that a significant proportion of cervical adenocarcinomas are not universally associated with HPV infection. According to the last WHO and International Endocervical Criteria and Classification (IECC) [45], gastric, mesonephric, clear cell, serous carcinoma, endometrioid adenocarcinoma, and adenocarcinoma NOS are HPV-unassociated carcinoma types. Then, in the current HPV vaccination era, the relative incidence of HPV-negative adenocarcinomas and their precursors may increase. Hypotheses of contributing factors to this increase in adenocarcinoma may be [46]:

- The relative reduction in cases of squamous lesions as a result of organized or opportunistic screening and HPV vaccine implementation;
- the impact of processing techniques (liquid-based cytology) modifications on cytodiagnosis and increasing of the proportion of AGC cervical smears;
- a change in the distribution of HPV types coupled with better recognition of glandular lesions by the pathologists;
- a higher prevalence of non-HPV vaccine genotypes infection in time.

The described relative as well as absolute increase in cervical adenocarcinomas, in the post-vaccination era, represent a further and new challenge that may increase difficulties in the colposcopic evaluation so that higher quality colposcopy and expertise will be necessary. Modeling studies indicate that the full impact of HPV vaccination on real-life data (HPV prevalence, preneoplastic lesions, and invasive tumors) might be registered in decades, but first in the youngest age groups. Regarding the natural history of adenocarcinoma, cervical cytology screening is more efficient for detection of CIN than adenocarcinoma in situ (AIS). AIS often goes undetected: 30–60% of AIS lesions are detected incidentally during treatment or follow-up for squamous abnormalities. Age at diagnosis for AIS is older than for CIN3; then longer time for the HPV vaccine to have a large impact on AIS incidence will be needed [47]. Cleveland et al. reported a significant decrease in AIS incidence rates among women screened for cervical cancer from 2008 to 2015 in the 21–24 year age group (annual percent change [APC] overall: −22.1%, 95% CI: −33.9 to −8.2; APC among screened: −16.1%, 95% CI: −28.8 to −1.2), decrease not observed in any older age group suggesting an important opportunity for vaccine prevention of AIS among young women during the vaccine era [47]. Vaccination could have impacted AIS rates directly, by preventing the HPV infections that could have progressed to AIS, or indirectly through a reduction in squamous abnormalities leading to fewer opportunities to incidentally detect AIS. Moreover, since HPV vaccine introduction in the USA, an ecological study, using population-based cancer registry data, reported that cervical squamous cell carcinoma decreased by 22.5% per year (during 2010–2017), and adenocarcinoma incidence rates declining 9.4% per year (during 2006–2017) among women aged 15–20 years. The observed reduction among very young women aged 15–20 years, a group not typically screened for cervical cancer, may suggest a relevant HPV vaccine impact [13]. 

### 3.4. Impact of Primary and Secondary Prevention on Colposcopic Practice

The impact that the combination of primary and secondary prevention has already had on colposcopic practice can be at least summarized into some features:

#### 3.4.1. Colposcopic Features and HPV Genoptypes

Is the appearance of the cervical lesion associated with the HPV genotype/s present? If so, HPV vaccination may alter the colposcopic features and thereby potentially affect the colposcopic practice. Prior studies have stated that colposcopic findings may vary according to HPV types. In particular, lesions related to type 16 were described as detected at a younger age, more definitive, and of larger size than other viral-type lesions [48,49,50,51]. In a population following European screening practices, HPV16-related CIN2+ lesions were detected at a younger age and showed similar colposcopic impression as non-HPV 16 HR high-grade lesions, but the PPV of colposcopy for any grade of abnormality and the high-grade impression was higher in HPV 16 positive women with CIN2 than in HPV 16 negative women [52]. The impact of genotypes on colposcopic features may also vary according to age [53]. It is likely that over the years we are already facing more lesions caused by other than HPV 16 and 18 types (OHR HPV), lesions described as smaller in size, less definitive, easier to miss, and, therefore, make the diagnosis less easy on initial colposcopy, or that appear more slowly and in older women or with low-grade cytology [54,55,56].

#### 3.4.2. Reduction of HPV Vaccine-Types Disease and Influence on Colposcopic PPV for CIN2+

Even if the HPV vaccine did not show a statistically significant effect on commonly recognized colposcopic features, there was a slight, non-significant reduction in the positive predictive value (PPV) of colposcopy for CIN2+, from 74% (unvaccinated) to 66.7% (vaccinated). The presence or absence of HPV 16 had a significant impact on the specificity and NPV of colposcopy for detecting CIN2+. HPV vaccination, therefore, reduced the prevalence of HPV 16 infection and HPV 16 CIN2+ and reduced the PPV of colposcopy for the detection of CIN2+ in vaccinated women [39]. Against the significant decrease in the colposcopic positive predictive value (PPV) for HSIL (CIN2+) on histology was reported in young Scottish women aged 20–21 years, from 79% in 2008/9 to 67% in 2013/14. In settings with both primary and secondary prevention, this impact of the vaccine on the key performance indicator of colposcopic accuracy points clearly that we are facing important changes to colposcopic practices [57]. As a matter of fact, there is a common opinion that monitoring colposcopy performance will become even more critical in the future as cervical cancer screening and primary prevention strategies evolve. Therefore, a higher-quality colposcopy service is an essential component of future cervical cancer screening programs. 

#### 3.4.3. Multiple Infections and Colposcopic Accuracy

In cases of cervical lesions caused by multiple HPV types, the reduction in the prevalence of HPV 16 will likely affect the timing of onset and size of the lesions. As the prevalence of HPV 16 and subsequently of other vaccine HPV genotypes decreases, we expect evidence of these changes, first of all, in vaccinated women. In routine clinical practice, multiple infections or HPV16 positivity status does not influence the colposcopic accuracy in the diagnosis of CIN3+ lesions. Among women with ASC-US/LSIL, assuming any colposcopic abnormality as a cut-off, there were no significant differences in the sensitivities (83.8%, 95% CI = 76–89.6 as compared to 84.1%, 95% CI = 73.2–91.1, *p* = 0.9) in the detection of CIN3+ lesions between subjects with single and multiple high-risk infection, and between subjects infected by HPV 16 (83.1%, 95% CI = 73.7–89.7, ROC = 0.59, 95% CI = 0.54–063) or other high-risk HPV (84.7%, 95% CI = 75.6–90.8, ROC = 0.62, 95% CI = 0.58–0.66, *p* = 0.8 and *p* = 0.6 compared to HPV16) [49]. Then, among CIN lesions diagnosed by LEEP or by cold-knife excisional procedures, the rates of multiple (≥2) as compared with single HPV infections increased from 31.7% (59/186) in smaller lesions (covering 0% to 25% of the cervix) to 39.2% (40/102), 41.9% (13/31), and 48.9% (45/92) in those covering 26% to 50%, 51% to 75%, and more than 75% of the cervical surface, respectively (χ^2^ for trend = 7.9; *p* = 0.005). Thus, multiple, as opposed to single HR HPV infection, is associated with larger colposcopic lesions expressed as a percentage of cervix surface covered by intraepithelial lesions diagnosed by excisional procedures. This direct association between multiple viral infection and larger cervical lesions was confirmed among subjects infected by HPV 16 (OR = 2.45; 95% CI = 1.14–5.26; *p* = 0.02) and in CIN3+ lesions (OR = 2.43; 95% CI = 1.23–4.80; *p* = 0.01) [51]. 

#### 3.4.4. Reduction in Number and Modification of Reasons for Referral to Colposcopy 

The vaccine administration has reduced over time the rate of HPV 16/18 and other than HR HPV 16/18 premalignant lesions. Considering the pattern of referral to colposcopy for abnormal screening tests, the proportion of LSIL increases with the corresponding decrease in the proportion of HSIL, and, therefore, the number of referrals to colposocpy for HSIL decrease over the observation period [57].

#### 3.4.5. Modifications of Colposcopic Performance and Procedures

The rates of diagnostic and therapeutic procedures following colposcopic examination underline the significant modifications that an evolving practice requires: major knowledge, expertise, and skills.

- Increase in cases with absence of abnormal colposcopic features [i.e., no acetowhite, no capillary vessel patterns (mosaic and/or punctation), or no abnormal vessels];
- increase in the proportion of women having no clinical interventions (biopsy or treatment);
- decrease in the proportion of women with a high-grade CIN colposcopic impression;
- decrease in the proportion having diagnostic punch biopsy/biopsies or treatment (loop excision or cold coagulation) [58].

### 3.5. Strategies to Preserve the Diagnostic Accuracy of Colposcopy in the Vaccination Era

**Figure 1:**
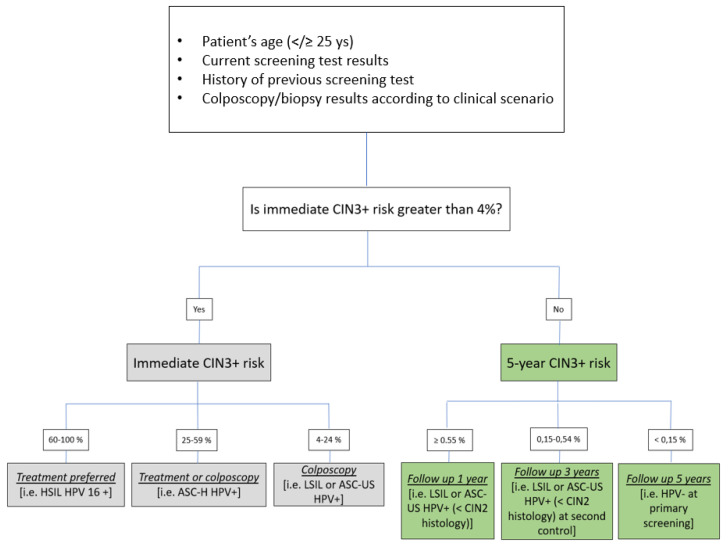
Management proposal based on patient risk of HSIL (CIN3+) stratification (16, 23).

In this context, and with this evidence, what could and should be done to maintain a good diagnostic accuracy of colposcopy in the vaccination era? First of all, further reduction in the inter- and intra-observer variability of the colposcopic practice including a less subjective and more objective patient selection. Artificial intelligence represents a promising tool that could help in obtaining needed standardization. Patient selection could be based on immediate or future risk of HSIL+ (CIN3+) stratification after a positive screening in different ways, as has recently been proposed by the ASCCP new guidelines [17] (Figure 1):

(A) Risk stratification based on age (</≥25), vaccination status, current test results, and history of previous screening test and colposcopy/biopsy results and expressed in a percentage ranging from 0.15 to 100%;
(B) risk groups based on the positivity of the single HR HPV genotype found adding partial or extensive genotyping to all positive HR HPV screening test cases;
(C) combination of A + B.

### 3.6. Applying Risk-Based Stratification to the Italian Screening Setting

In settings like Italy, the applicability of the aforementioned guidelines and proposals is however premature and should, in any case, be preceded by adequate clinical validation and by adaptation of the risk stratification process to the Italian population and careful consideration of the possible legal consequences that could be derived from it. What would it mean to apply risk stratification to current Italian screening that is based on cytology or HR HPV test according to age? An application is already available online to calculate the risk of CIN3+ for each patient and at each visit on a tablet or smartphone. How could this new approach change by adding extended HPV genotyping? In screened women with less than 30 years with ASC-US or LSIL Pap smear would it mean adding a full genotyping to all, then referring directly to colposcopy only those positive for higher risk for CIN3+ HPV (16, 31, and 18) or even those positive for 33, 45, 52, and 58? Currently, in young Italian cohorts, the HPV test is not used as a screening test. On the other hand, after the age of 30, all women positive for HR HPV screening test would be subjected to full genotyping, those positive at 16, 18, and 31 referred directly to colposcopy, those positive for another 11 HR HPV to cytology. Of those, the HSIL cytologies would clearly need direct colposcopy. Even cytologies ASC-US/LSIL positive for 33, 45, 52, and 58 HPVs would be referred to colposcopy, while other low-grade cytologies positive for 35, 39, 51, 56, 59, 66, and 68 would go to control after 1 year such as negative cytologies. However, we are aware that any change to the current protocols would take time, validation through scientific data, and discussion within accredited societies. We are also convinced that this policy would indeed mean not only a big change in colposcopy practice, first of all in follow-up timing modifications and delayed referral to colposcopy for ASC-US/LSIL cases positive for lowest risk HPV group genotypes (35, 39, 51, 56, 59, 66 and 68), but also less costs and less patient anxiety. A stratification of each patient’s own risk of CIN3+ at screening could help towards tailored management [14,22,23,24]. Recently, the Polish Society of Colposcopy and Cervical Pathophysiology and the Polish Society of Gynecologists and Obstetricians in fact provided clinical consensus guidelines for colposcopy practice in secondary cervical cancer prevention including the vaccination status into the optimal protocol of colposcopy practice. To enhance the standard of colposcopy, adjustment of a precolposcopic assessment, a performance technique, types of biopsies, as well as the procedure documentation were made. Even the HPV vaccination status was included as an element of the risk-based stratification for the increased risk of developing cervical cancer. In fact, the pre-colposcopy evaluation options were divided into basic (with seven recommended parameters to consider) and optimal (with 19 parameters). Only in an optimal protocol are the vaccination status, the name of the vaccine, and the number of doses considered as recommended parameters [59]. We actually need data to support alternative colposcopic strategies based on risk stratification and vaccination status. The personal risk stratification is implicit in precision medicine and from this perspective the histopathological diagnosis until now is considered the “gold standard” of an individual disease, is only a piece of “the diagnostic puzzle” where other pieces consist of HR HPV positivity/genotype/types, cytologic, colposcopic, histological and microbiota reports, vaccination status, and other genetic and epigenetic factors. In the vaccination era, the study of the vaginal microbiota has revealed important correlations between vaginal dysbiosis and microbiota composition and the natural history of HPV disease as well. In fact, the recent literature reports an association between certain bacterial community types of the vaginal microbiota—microbiota vaginotype—and HPV infection, persistence, and HPV-related disease. Moreover, the presence of specific anaerobic species and Lactobacillus depletion at the time of CIN2 diagnosis was associated with a significantly lower chance of regression at 12- and 24-month follow-up. In clinical management, these results will undoubtedly be somehow precious as biomarkers for HPV-related disease, guiding treatment options and follow-up of untreated and treated patients [59,60,61].

## 4. Conclusions

The strategies of cervical carcinoma prevention are continuously changing even with differences in the modalities and timing between the various real-world scenarios. At the moment, the synergy between primary and secondary screening is strong and is obtaining satisfactory results. There is a need for innovative approaches to cervical cancer eradication and, in this context, current efforts are focused on the continuous search for new or better screening tests and for maximum vaccination effectiveness. The Italian Society of Colposcopy and Cervico-Vaginal Pathology (SICPCV) is committed to supporting vaccination programs for children, adolescents, young adults, and post-treatment for HSIL together with a catch-up vaccination [62,63,64]. In Italy, there is a strong colposcopic tradition, and clinical colposcopic practices are continuously transmitted from one generation to the next. They are taught to postgraduate residents in Graduate Schools in Obstetrics and Gynecology and to gynecologists working in public and private facilities attending regional and national societies of colposocpy (SICPCV) accreditation courses. Moreover, the costs of a colposcopic examination in Italian public health services are much lower than in private healthcare while all services are free of charge within the organized screening program. The introduction of HPV vaccination as well as the switch to HPV-based screening is changing the profiles of women presenting to colposcopy services and provide management difficulties for the colposcopist. The question is: “May the improvements in the performance of cervical screening be limited by the diagnostic performance of colposcopy in the vaccination era”? We are making every effort to maintain diagnostic accuracy and the informational role of colposcopic examination. Until now, the morphological approach, as a whole, in the prevention of cervicocarcinoma represented the diagnostic gold standard (cytology, colposcopy, histology). We are moving from operator-dependent morphology-based screening/diagnosis/treatment to reduced operator-dependent and towards biomolecular management. The introduction of risk stratification based on any combination of “patient’s history”, “vaccination status”, and “test results” to decrease colposcopy referral is further reducing the colposcopic and histologic morphological approach while the introduction of genotyping in the risk stratification paradigm means less cytologic morphologic diagnosis. Indeed, in cases of co-testing as the primary test or HPV primary test with triage citology, the simultaneous availability of both tests allows a more real risk stratification. Furthermore, it is mandatory to consider other aspects such as lost-to-follow-up, false-negative-tests, different individual risk factors involved in carcinogenesis, and other major themes of counseling. In the perspective of precolposocpic assessment risk stratification, one might speculate the possible role of screening test transition from the current primary HR HPV test to a partial or full genotyping test that could allow initial risk stratification without significantly increasing costs. The future goal of the ongoing revolution is the limitation of colposcopic practice, an expensive and subjective instrumental examination, that seems achievable through the impact of vaccination on HSIL prevalence and patient’s risk-stratified approach to direct referral. Moreover, as in other fields of medicine, we are faced with ongoing research for balancing maximal effort and minimum costs. At the moment, this somewhat necessary process appears as a rather innovative and more complex approach, the eventual introduction of which should be modulated and capillary adapted to every national and regional reality. We rather deem that there is currently no immediate need to reduce the number of colposcopies in Italian screening programs, unlike what occurs in some countries, where the cost of the entire colposcopy workup is much higher, where healthcare is mainly private, and where limiting this practice has another purpose and different meaning. We believe that the actual second-level Italian cervical cancer screening is satisfactory and adequate even if further updating and improvements are needed. In particular, there is a need for more accredited colposcopists. It has become essential to focus on some critical issues belonging to yesterday, today, and to the future post-vaccinal era: maintain the diagnostic accuracy of colposcopy, manage the persistence of HR HPV, screen and diagnose non-HPV related cervicovaginal lesions and adenocarcinoma of the cervix. We also believe that the interaction between the cervicovaginal microbiota and both the persistence of HR HPV and the progression of lesions of the lower genital tract already represents a risk factor and, among well-known researchers, will undoubtedly have an important role in the clinical management of our patients in the future (Box 4).

Box 4 **Colposcopy and future perspectives.**

- Colposcopic practice is changing in the HPV vaccination era;
- high-quality colposcopy service remains an essential component of future cervical cancer screening programs;
- the colposcopist remains the clinical manager;
- colposcopic guidelines are moving towards patient risk stratification;
- risk stratification process will gradually include other factors (overage, current and previous tests, viral genotype) such as vaccination status, patient’s immune health status, microbiota vaginotype, genetic and epigenetic factors available in clinical evaluation, etc.;
- the reduction in colposcopic PPV is a point of concern; colposcopy will increasingly become an expert investigation;
- future strategies to maintain and increase expertise and informational role of colposcopy;
- the role of national and international Societies of colposcopy remains fundamental; national societies of colposopy should provide continuous, updated, available, and mandatory accreditation to all colposcopic professionals;
- application of artificial intelligence models to colposcopic practice to improve diagnostic accuracy can be the future.

## Data Availability

Not applicable.
